# Virtual auscultation course via video chat in times of COVID-19 improves cardiac auscultation skills compared to literature self-study in third-year medical students: a prospective randomized controlled cross-over study

**DOI:** 10.3205/zma001542

**Published:** 2022-04-14

**Authors:** Nils Rüllmann, Raphael Hirtz, Unaa Lee, Kathrin Klein, Ertan Mayatepek, Bastian Malzkorn, Carsten Döing

**Affiliations:** 1 Heinrich-Heine-University Düsseldorf, Medical Faculty, Office of the dean of studies, Düsseldorf, Germany; 2 University Children's Hospital Düsseldorf, Department of General Pediatrics, Neonatology and Pediatric Cardiology, Düsseldorf, Germany; 3 University of Duisburg-Essen, Department of Pediatrics II, Division of Pediatric Endocrinology and Diabetology, Essen, Germany; 4 University Hospital Düsseldorf, Division of Cardiology, Pneumology and Angiology, Düsseldorf, Germany

**Keywords:** virtual auscultation, video chat, literature self-study, distance-based learning, patient simulator, heart auscultation, COVID-19, skills lab, peer teaching

## Abstract

**Background:**

Cardiac auscultation is a core clinical skill taught in medical school. Due to contact restrictions during the SARS-CoV-2 pandemic, interaction with patients was very limited. Therefore, a peer-to-peer virtual case-based auscultation course via video conference was established.

**Methods:**

A randomized controlled cross-over study was conducted to evaluate whether participation in a virtual auscultation course could improve heart auscultation skills in 3^rd^-year medical students. A total of sixty medical students were randomly assigned to either the experimental or control group after informed consent was obtained. Due to no-shows, 55 students participated. Depending on allocation, students attended three ninety-minute courses in intervals of one week in a different order: a virtual case-based auscultation course held via video chat, literature self-study, and an on-site course using a high-fidelity auscultation simulator (SAM II). The study's primary endpoint was the performance of the two groups at the simulator after participating in the virtual auscultation course or literature self-study. To evaluate their auscultation skills, students participated in five assessments using the same six pathologies: stenosis and regurgitation of the aortic and mitral valve, ventricular septal defect, and patent ductus arteriosus. Moreover, participants rated their satisfaction with each course and provided a self-assessment of competence.

**Results:**

Compared to literature self-study, participation in the virtual auscultation course led to a significantly improved description of heart murmurs at the auscultation simulator with regard to the presence in systole and diastole, low- and high-pitched sounds, and volume dynamics. There was no significant difference between the groups in diagnostic accuracy and identification of the point of maximal intensity. After the virtual course, students showed higher satisfaction rates and a higher increase in self-assessed competence compared to participants who engaged in literature self-study.

**Conclusions:**

For the first time, this study demonstrates that a case-based virtual auscultation course can improve aspects of cardiac auscultation skills on a simulator. This may facilitate the further acquisition of an essential clinical skill, even when contact restrictions will be lifted.

## Zusammenfassung

**Hintergrund:**

Die Herzauskultation ist eine der wichtigsten klinischen Fertigkeiten, die im Medizinstudium gelehrt werden. Aufgrund von Kontaktbeschränkungen während der SARS-CoV-2-Pandemie war die Interaktion mit Patienten sehr begrenzt. Daher wurde ein Peer-to-Peer-Kurs zur fallbasierten Auskultation per Videokonferenz entwickelt.

**Methoden:**

In einer randomisierten, kontrollierten Cross-over-Studie wurde untersucht, ob die Teilnahme an einem virtuellen Auskultationskurs die Herzauskultationsfertigkeiten von Medizinstudierenden im dritten Studienjahr verbessern könnte. Sechzig Medizinstudierende wurden randomisiert einer Experimental- oder Kontrollgruppe zugeteilt. Aufgrund von Nichterscheinen nahmen insgesamt 55 Studierende teil. Je nach Zuteilung besuchten die Studierenden in unterschiedlicher Reihenfolge drei neunzigminütige Kurse im Abstand von einer Woche: einen virtuellen, fallbasierten Auskultationskurs per Videokonferenz, ein Fachliteratureigenstudium und einen Kurs mit einem High-Fidelity-Auskultationssimulator (SAM II) in Präsenz. Der primäre Endpunkt der Studie war die Performance der beiden Gruppen am Simulator nach der Teilnahme am virtuellen Auskultationskurs oder am Fachliteratureigenstudium. Um ihre Auskultationsfähigkeit zu bewerten, nahmen die Studierenden an fünf Assessments teil, bei denen stets dieselben sechs Pathologien verwendet wurden: Stenose und Insuffizienz der Aorten- und Mitralklappe, Ventrikelseptumdefekt und persistierender Ductus arteriosus. Außerdem bewerteten die Teilnehmenden ihre Zufriedenheit mit den einzelnen Kursformaten und gaben eine Selbsteinschätzung der Zunahme ihrer Kompetenz ab.

**Ergebnisse:**

Im Vergleich zum Fachliteratureigenstudium führte die Teilnahme am virtuellen Auskultationskurs zu einer signifikant verbesserten Beschreibung der Herzgeräusche am Auskultationssimulator in Bezug auf Zuordnung zu Systole und/oder Diastole, Identifizierung hoher und tiefer Frequenzen sowie Lautstärkedynamik. Bei der diagnostischen Präzision und der Identifizierung des Punctum maximum gab es keinen signifikanten Unterschied zwischen den Gruppen. Nach dem virtuellen Kurs zeigten die Teilnehmenden eine höhere Zufriedenheit und einen höheren Zuwachs an selbst eingeschätzter Kompetenz im Vergleich zu den Teilnehmenden des Fachliteratureigenstudiums.

**Schlussfolgerung:**

In dieser Studie wurde erstmals nachgewiesen, dass ein fallbasierter virtueller Auskultationskurs Aspekte der Herzauskultation am Simulator verbessern kann. Dies kann den weiteren Erwerb dieser wesentlichen klinischen Fertigkeit erleichtern, auch wenn Kontaktbeschränkungen aufgehoben werden.

## 1. Background

Cardiac auscultation using a stethoscope allows immediate identification of important cardiac pathologies. It is easily accessible and part of most physical examinations enabling skilled examiners to make an accurate diagnosis [1] or to initiate further diagnostics.

Auscultation is an essential examination technique. However, it requires the development of complex auditory skills. In medical education, these skills are taught through lectures, examination courses, and bedside teaching. Bedside teaching allows medical students to learn in a realistic environment, place heard heart sounds in context with other physical findings and recognize typical patterns of sound radiation across the thorax. However, infrequent exposure to rare findings as well as a high ratio of learners to patients pose challenges [2]. Moreover, the variability of clinical presentations prevents standardized teaching [3], and patients (e.g., young children) may not be amenable to or available for examination, most recently due to contact restrictions during the SARS-CoV-2 pandemic.

In the past, many studies have shown poor auscultation skills of medical students [4], residents, as well as practicing physicians [4], [5], [6], [7], [8], illustrating the need for better training methods. Although a core clinical skill, clinical practice does not necessarily improve cardiac auscultation skills [9], [10], [11].

Addressing a generation of digital natives, the use of simulation technology and e-learning for skills training and assessment in medical education has progressively increased over the last two decades: Multiple simulation methods to train cardiac auscultation, summarized in a review of Ward and Wattier [12] and a meta-analysis by McKinney, Cook [13], allow for improving the transfer of cardiac auscultation skills to clinical settings [14]. Virtual teaching programs range from sound simulation [15], [16], [17] to virtual patient examinations [18] and pediatric e-learning courses on cardiology basics [19]. Auscultation training on on-site simulators (e.g., Student Auscultation Manikin=SAM II) and simulated patients in skills labs provide another opportunity to learn and improve auscultation skills of normal and pathological findings [2], [12], [14], [20], [21], [22], [23], [24], [25], [26].

During the ongoing contact restrictions due to the SARS-CoV-2 pandemic, courses at the on-site skills lab with an auscultation manikin were suspended, and interaction with real patients in the context of bedside teaching was compromised [27]. Similar obstacles were described during the outbreak of SARS-CoV-1 [28]. The urgent need for alternative training methods turned out to be an opportunity to further catalyze the modernization of medical education [29] at our faculty during the summer term of 2020: A virtual auscultation course (VAC) via video conference was designed by a student with extensive experience in peer-teaching and supervised by specialist cardiologists [27].

According to results from previous studies [14], [20], [21], [22], [23], the following hypotheses (H) were derived regarding the effect of a VAC on the auscultation skills of medical students: Participation in the VAC allows for better description (H_1_), higher diagnostic accuracy (H_2_), and more reliable identification of the point of maximal intensity (H_3_) of heart murmurs when compared to literature self-study, immediately after the course (H_1A_, H_2A_) and at follow-up at the simulator (H_1B_, H_2B_, H_3_). The participant’s performance at the simulator was the study’s primary endpoint (H_1B_, H_2B_, H_3_).

To allow participants to benefit from all teaching methods, a cross-over design was chosen. It was hypothesized, that this approach results in comparable cardiac auscultation skills in participants by the end of the study, irrespective of the randomization order (H_4_) [30], [31].

Following previous evaluation results [27], it was expected that attendance of the virtual auscultation course leads to increased satisfaction (H_5_) and a higher increase in self-assessed competence (H_6_) when compared to literature self-study.

## 2. Methods

### 2.1. Study design

A prospective, randomized, controlled cross-over study was carried out at the Medical Faculty of the Heinrich-Heine-University of Duesseldorf, Germany. The study was designed to compare cardiac auscultation skills after participating in a one-time ninety-minute, virtual case-based auscultation course with participation in time-equivalent literature self-study.

**Figure 1:**
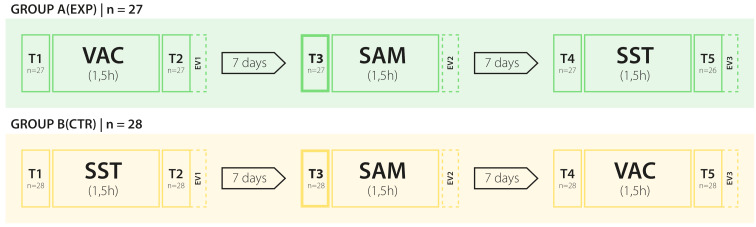
Study design: a prospective controlled cross-over design including three interventions (VAC: virtual auscultation course, SAM: student auscultation manikin, SST: literature self-study), five assessments (T1-T5) and evaluations (EV) of student's satisfaction and increase in self-assessed competence after every intervention (EV1-EV3) one week apart. Order of exposed group in green, order of control group in yellow. The participant's performance at T3 is defined as the primary endpoint.

As shown in figure 1 (Fig. 1), three course formats were offered during this study in a different order: a virtual auscultation course (VAC), literature self-study (SST) during a video conference, both held via Microsoft Teams (MS Teams; [https://www.microsoft.com/de-de]), and an on-site course using a student auscultation manikin (SAM). Courses took place one week apart in groups of 5 (SAM) to 10 participants (VAC & SST).

Auscultation performance was assessed on five occasions (T1-T5) using the same six pathologies found in adult and pediatric patients: stenosis and regurgitation of the aortic and mitral valve, ventricular septal defect, and patent ductus arteriosus. The primary endpoint of the study was the performance at an auscultation simulator seven days after participating in one of two different interventions. Group A (exposed) participated in a virtual auscultation course (VAC), Group B (Control group) in literature self-study. Assessments T1, T2, T4, T5 were conducted before and after the VAC and SST to monitor any direct effect on the auscultation performance.

Participant's satisfaction and self-rated competence were assessed using evaluation forms completed after every course (EV1-EV3).

Approval for the study was obtained from the Ethics Committee of the Medical Faculty, Heinrich Heine University, Germany, (Nr. 2021-1298). The study was conducted in accordance with the Declaration of Helsinki.

#### 2.2. Randomization

**Figure 2:**
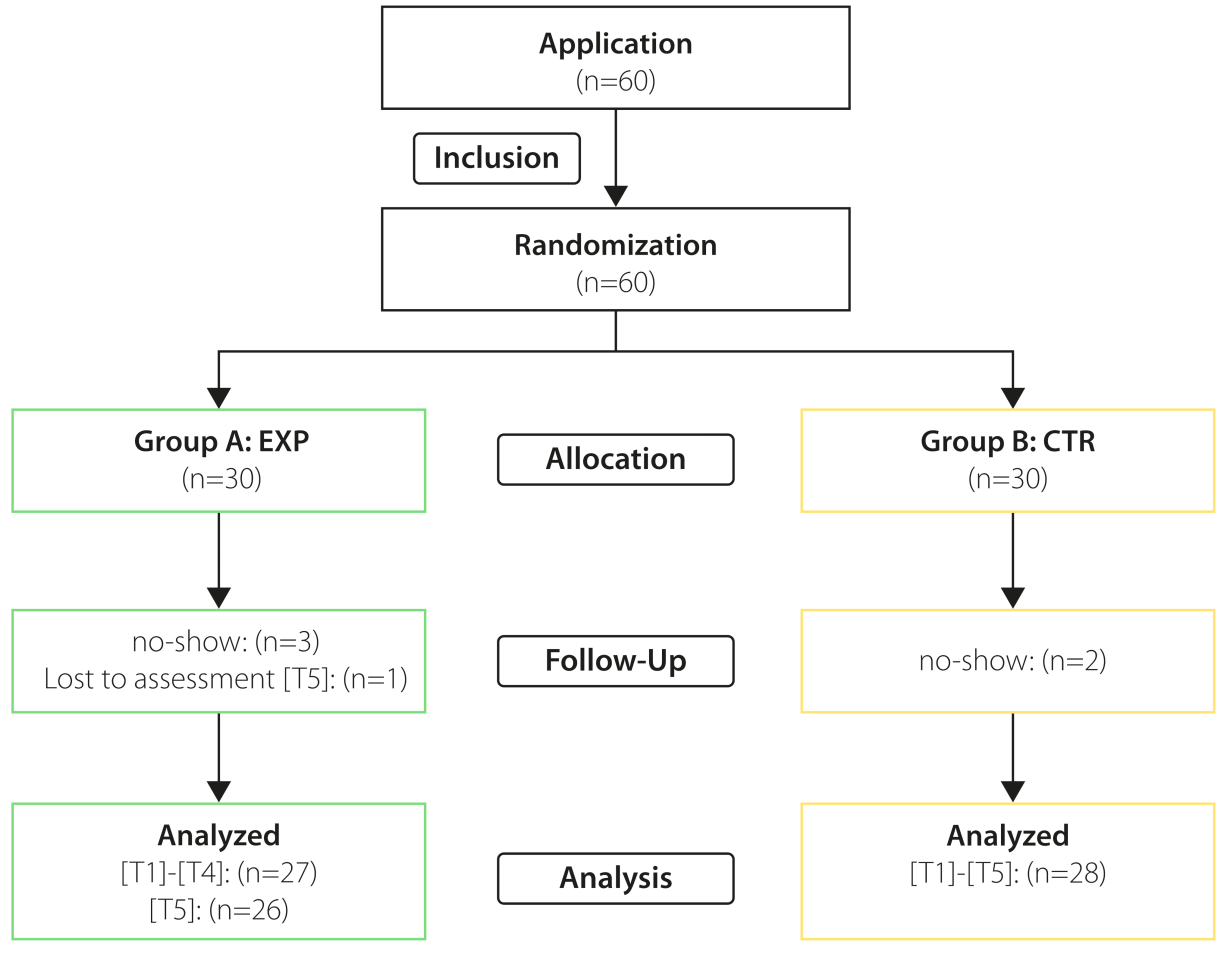
Randomization: N = 60 medical students were recruited and randomly assigned to two equally sized groups using random numbers generated by the Research Randomizer [https://www.randomizer.org]. Group A (EXP) = exposed group, B (CTR) = control group. Due to no-shows, the study started with n=55 participants (A: n=27; B: n=28); one participant of group A was lost to follow-up. Inclusion criteria: Age > 18 years, third year medical students at Heinrich Heine University Düsseldorf, internet access, declaration of consent to participate in the study. Exclusion criteria: Withdrawal of consent.

Sixty third-year medical students were recruited on a voluntary basis and provided informed consent to participate. Successful attendance was rewarded with 10€. The randomization process is shown in figure 2 (Fig. 2). During the first two years of the curriculum, students at the Medical Faculty of the Heinrich Heine University participate in a preparatory course for internships, learning cardiac auscultation of the healthy heart. Pathological findings are covered during cardiology lectures in the fourth year of their studies.

#### 2.3. Interventions

Students participated in three courses (VAC, SST, SAM) of equal length (90 min) in two different orders, depending on randomization. To avoid bias, all courses were held by the same tutor.

##### 2.3.1. Virtual auscultation course (VAC)

The interactive online seminar was designed to improve listening technique, description, and interpretation of auscultation findings in an off-site context [27]. This case-based course was held by an experienced medical student as peer-teaching has proven its benefit for both teachers and learners [30], [32]. Clinical cases with synthesized auscultation sounds were presented adapted to the model of case-based learning [33], [34]. Adult and pediatric cases were discussed.

With kind permission, synthesized heart sounds, provided by the online learning program Clinisurf [https://clinisurf.elearning.aum.iml.unibe.ch/] developed at the University of Bern, Switzerland, were used.

##### 2.3.2. Literature self-study (SST)

The control intervention consisted of studying excerpts from two German textbooks teaching cardiac auscultation: “Füeßl: Anamnesis and Clinical Examination” ([35], p.184, 196, 197-207) as the reference of the medical faculty’s internship preparatory course and “Erdmann: Clinical Cardiology” ([36], p.375-377, 381-383, 397-415) as a reference textbook for cardiology lectures. These excerpts were made available as eBook chapters at the beginning of a supervised, 90 minutes video conference. The description of heart murmurs, including phonocardiograpic visualizations, and the presented clinical knowledge corresponded to that of the VAC.

So far, there is no evidence on the efficacy of literature study on cardiac auscultation. It was chosen as control intervention because it represents a standard method for medical students to prepare for the clinical environment. Self-study is thus a relevant comparator even if teaching auscultation via literature alone is uncommon and the effectiveness of self-study has not been evaluated previously.

##### 2.3.3. Auscultation simulator (SAM)

In the skills lab, participants auscultated the simulator Cardionics Student Auscultation Manikin II (SAM, Cardionics, Webster, Texas, USA) which presented the same six heart murmurs. Auscultation performance was assessed (T3). After the assessment, different heart murmurs were presented, and associated cardiac pathologies and their clinical background were discussed with the tutor.

#### 2.4. Assessment

The order of the cardiac pathologies was randomized using the Research Randomizer [https://www.randomizer.org/]. To avoid confounding, the order of pathologies was identical following the study's cross-over design: Before VAC, SST and SAM and after VAC and SST, the same order was used for both groups.

Directly before and after VAC and SST (T1, T2, T4, T5), students listened to auscultation sounds using their own headphones, lasting 15 minutes. They were asked to describe the sound characteristics (e.g., systolic/diastolic murmur, low-/high-pitched sound, volume dynamics) and make a diagnosis of each auscultation finding. The presented auscultation files were those used in the VAC and were the same for the assessments T1, T2, T4, T5. The participants did not receive feedback.

At the simulator, participants used their own stethoscopes to auscultate the same murmurs studied during the VAC and SST. In addition to a description of sound characteristics and a diagnosis regarding the presented pathologies at T1, T2, T4, T5, participants were also asked to indicate a point of maximal intensity for each heart murmur. The synthesized auscultation sounds were provided by Cardionics, the manufacturer of the auscultation manikin SAM II.

#### 2.5. Data collection & processing

For the performance categories “sound description” (DESC), “diagnosis” (DIAG), and, at the simulator, “point of maximal intensity” (PM), participants provided their answers via questionnaires in an open text format. The assessments were performed with the Medical Faculty's online evaluation form and anonymized by source. For online courses (VAC & SST), the questionnaires (T1, T2, T4, T5) were accessible via internet. Time stamps ensured the participant's compliance with the 90 minutes time frame directly before or after the courses. For the on-site course (SAM, T3), printed questionnaires were completed manually and digitalized later.

Answers on each item were compared to a list of accepted answers and assessed by two raters. In cases of disagreement, the final score was assigned by a third rater, a cardiologist. A correct answer was awarded one point. For the evaluation of results scores were separately summed for each of the assessed performance categories (DIAG, DESC, PM). The maximum score in each category was six points.

Additional evaluation forms (EV1-EV3) using six-point Likert scales (best score=1) were employed after every course to measure satisfaction and self-assessed increase in competence.

#### 2.6. Statistics

Data handling and statistical analyses were performed with SPSS 27 (Armonk, NY: IBM Corp.). H_1-6_ were assessed by two-tailed testing and results deemed significant at p<.05. Considering that H_4_ coincides with the (statistical) H_0_ (i.e., no performance difference at T5 between groups regarding different intervention sequences), H_4_ was assessed at an adjusted α-level of *p*<.20. This approach allows for increasing the chance to reject H_0_, as recommended when testing for equivalence [37]. All other analyses were deemed exploratory and therefore, not corrected for multiple comparisons.

Effect size was interpreted according to Cohen [38] (*d*: small 0.20≤d≤0.49, medium 0.50≤d≤0.79, large≥0.8). Power analyses were performed with GPower 3.1 [39] (assuming: α=.05, two-tailed testing, sufficient power=1-β≥0.8).

Sum scores regarding diagnosis and sound description at T1-T5 as well as identification of the point of maximal intensity at T3 were non-normally distributed and contained outliers as identified by Shapiro-Wilk tests, visual inspection of Q-Q plots, and boxplots. This also applied to the items evaluating course satisfaction and self-assessed competence (H_5_, H_6_). When also considering the design of the study that did not allow for a repeated measure approach, Mann-Whitney U tests were performed to compare the experimental and control group concerning all outcome measures. 

## 3. Results

### 3.1. Cardiac auscultation skills

**Figure 3:**
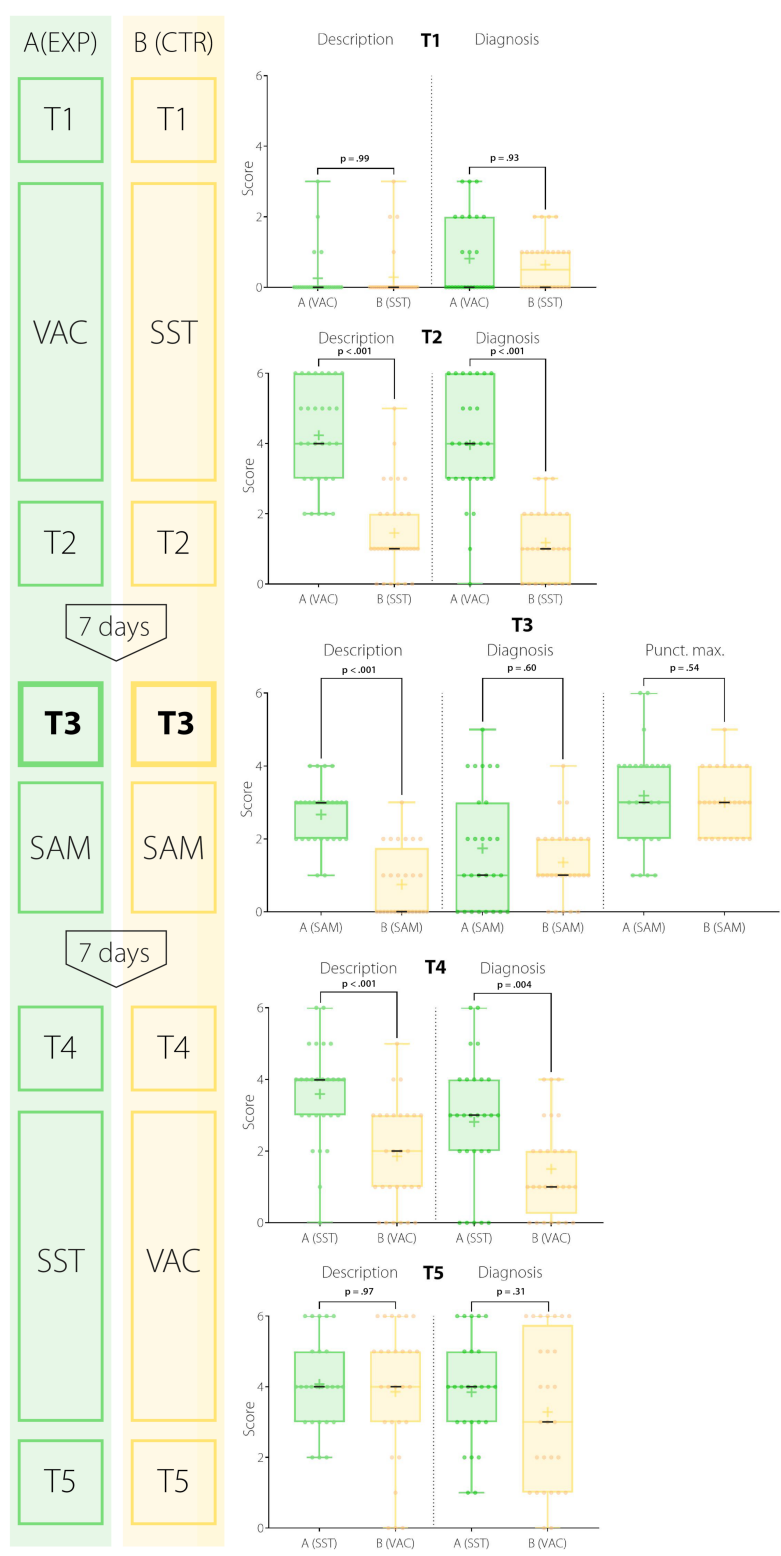
Cardiac auscultation performance: Sums scores of diagnostic accuracy, sound description at T1-T5 as well as identification of point of maximal intensity at T3 shown in bee-swarm boxplots (maximum score=6). Results of exposed group in green, results of control group in yellow. (+)=mean; (-)=median. Primary endpoint at T3.

There was a significant difference in description performance (DESC; e.g. systolic/diastolic murmur, low-/high-pitched sound, volume dynamics) between the experimental (N=27) and control group (N=28) immediately after the first course (T2: *Median_exp_*=4, *Median_control_*=1; *p*<.001, *d*=2.06), at the simulator (T3: *Median_exp_*=3, *Median_control_*=0; *p*<.001, *d*=2.06), and before the third course (T4: *Median_exp_*=4, *Median_control_*=2; *p*=<.001, *d*=1.24), which was not present at the first assessment (T1: *Median_exp_*=0, *Median_control_*=0; *p*=.99; H_1A-B_ proved), (see figure 3 (Fig. 3) and table 1 (Tab. 1)).

In terms of diagnostic accuracy (DIAG), no significant difference was found between groups at the first assessment (T1: *Median_exp_*=0, *Median_control_*=0.5; *p*=.93) and at the simulator (T3: *Median_exp_*=1, *Median_control_*=1; *p*=.60; H_2B_ disproved). However, immediately after the first course and before the third course, there was a significant difference (T2: *Median_exp_*=4, *Median_control_=1; p*<.001, *d*=2.05; T4: *Median_exp_*=3, *Median_control_*=1, *p*=.004, *d*=0.82; H_2A_ proved) between interventions.

Participation in the VAC did not allow for better identification of the point of maximal intensity of heart murmurs (PM) at the simulator (T3: *Median_exp_*=3, *Median_control_*=3; *p*=.54; H_3_ disproved).

Note, however, that in all sum score-related analyses, power was only sufficient for detecting a large effect size (power (1-β): *d*≥0.8=0.81).

#### 3.1.1. Evaluation of the cross-over design (H_4_)

At the final assessment (T5), no difference between the experimental (N=26) and control group (N=28) concerning DIAG and DESC was found (DESC: *Median_exp_*=4, *Median_control_*=4; p=.97; DIAG: *Median_exp_*=4, *Median_control_*=3; *p*=.31; H_4_ proved).

#### 3.2. Course satisfaction and self-assessed competence

**Figure 4:**
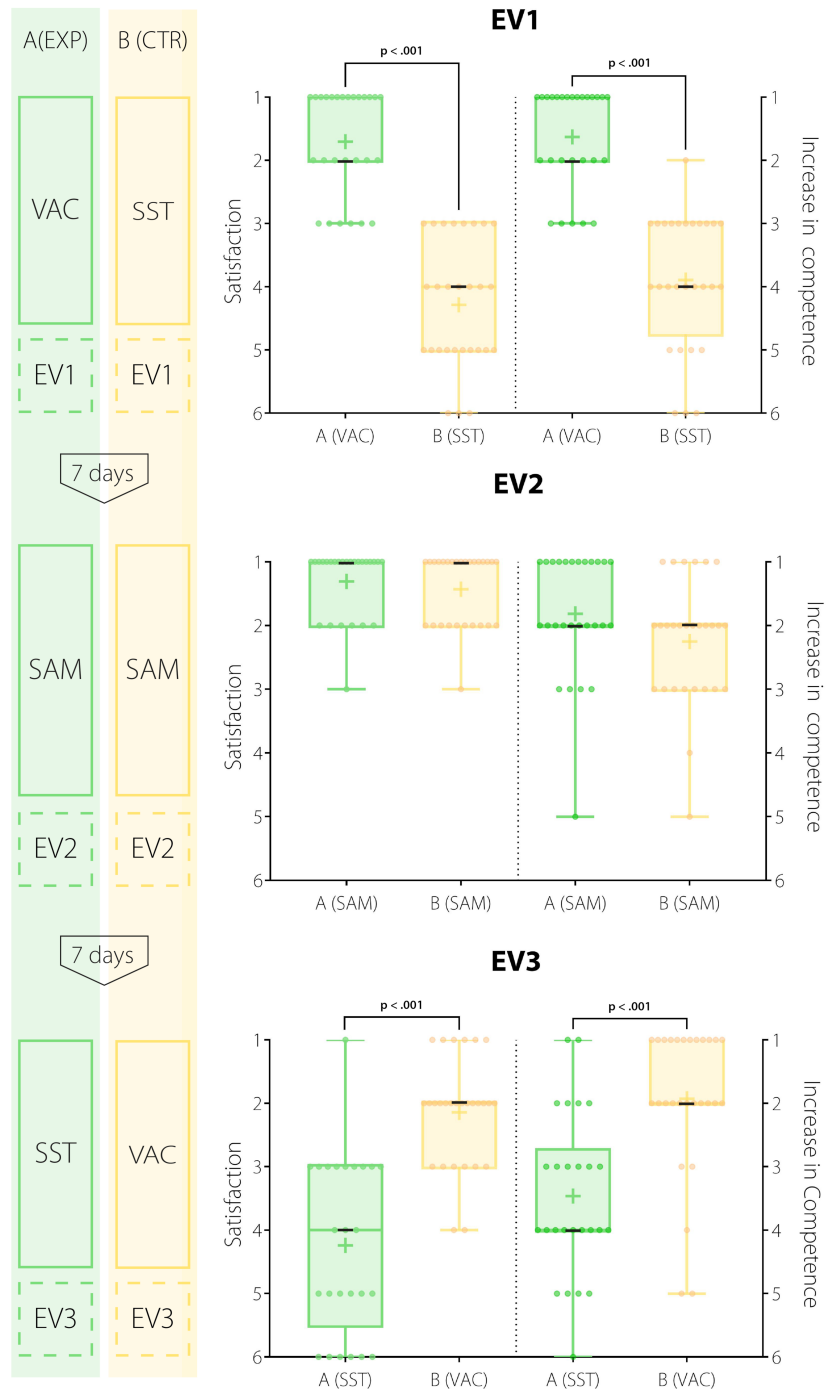
Evaluation results: Six-point Likert scale-based evaluation (best score=1) of participant’s satisfaction and self-assessed increase in competence after course participation in bee-swarm boxplots. Results of exposed group in green, results of control group in yellow. (+)=mean; (-)=median. No testing at EV2.

Participants of the VAC reported a significantly higher satisfaction compared to the literature self-study group (see figure 4 (Fig. 4) and table 2 (Tab. 2)). This was found in group A (EXP) after the first course (EV1: *Median_exp_*=1, *Median_control_*=4; *p*<.001,* d*=2.70) and in group B (CTR) after the third course (EV3: *Median_exp_*=4, *Median_control_*=2; *p*<.001, *d*=1.92; H_5_ proved).

Participation in the VAC resulted in significantly higher increase in self-assessed competence than participation in literature self-study. Group A (EXP) reported a higher increase after the first course (EV1: *Median_exp_*=1, *Median_control_*=4;* p*<.001,* d*=2.46), just like group B (CTR) after the third course (EV3: *Median_exp_*=4, *Median_control_*=2; *p*<.001, *d*=1.31; H_6_ proved).

At the auscultation simulator, both groups showed comparably high levels of satisfaction (EV2: *Median_exp_*=1, *Median_control_*=1) and indicated a high self-assessed increase in competence (EV2: *Median_exp_*=2, *Median_control_*=2).

## 4. Discussion

The present study demonstrates for the first time that a case-based virtual auscultation course via video conference may significantly improve the description of cardiac auscultation findings on a simulator compared to literature self-study.

### 4.1. Impact on study participants

Medical students participating in the VAC evidenced a distinct increase in the ability to describe sound characteristics of heart murmurs at the simulator. No significant difference in diagnostic accuracy was found. A similar effect was seen in a study by Giovanni et al. [24] comparing student's auscultation performance on patients six weeks after listening to auscultation files or training at an auscultation simulator. After training with a simulator, students showed better performance in heart sound interpretation without higher diagnostic accuracy.

Establishing an auscultation-based diagnosis is presumably linked to declarative, that is, explicit knowledge (e.g., “Aortic stenosis is a systolic murmur”). In contrast, the description of heart murmurs can be considered an implicit skill depending on procedural knowledge [40]. Unlike declarative knowledge, procedural knowledge is exercised in tasks. The results of the present study suggest that participation in the VAC results in superior retention of procedural than declarative knowledge. Importantly, in many instances, procedural knowledge may constitute the more important type of knowledge. As outlined by Kumar and Thompson [11] physicians do not necessarily require high diagnostic accuracy to identify a variety of different murmurs but should be able to distinguish physiological from pathological findings to initiate further diagnostics and cardiologist referral, if necessary.

In line with these considerations, a reliable acquisition and retention of procedural knowledge over a period of one week, as observed in the present study, has previously been reported by a study by H'Mida, Degrenne [41] comparing video and static images to teach a motor skill.

However, the observation that participation in the VAC resulted in the improved description of heart murmurs but not higher diagnostic accuracy may also be related to effects of guessing. There was a high variability in making the correct diagnosis at the simulator, mainly driven by some participants that made the right diagnosis without a correct description of the causal pathology.

Participation in the VAC did not significantly improve identification of typical patterns of sound radiation across thorax (PM). One possible explanation might be that the VAC did not allow to virtually auscultate all auscultation points on the thorax in each presented case, so participants had no opportunity to elaborate on the patterns of sound radiation individually.

#### 4.2. Implications for cardiac auscultation training

In this study, the VAC and training on a simulator led to high rates of satisfaction and a notable increase in self-assessed competence in third year medical students. This might lead to a greater benefit from further auscultation training (e.g. bedside teaching) during the participant's ongoing medical studies as previously suggested by Bernardi et al. [20], demonstrating the importance of early training of essential examination skills.

Multiple studies have shown positive effects of simulation-based auscultation training, ranging from sound simulation [15], [16], [17] to high-fidelity simulators [20], [21]. Simulators like Harvey or SAM provide standardized training featuring many aspects of real patients, but they come at a high price and rationed practice time [24]. During contact restrictions access is further limited.

Considering these limitations, video conference-based auscultation courses may be a valuable addition to the already existing repertoire of auscultation training methods. Participation in training offered face-to-face or by video conferences can lead to similar levels of knowledge and confidence in healthcare professionals [42]. This is confirmed by the results of the present study regarding cardiac auscultation skills in medical students.

Consequently, further research is needed to examine skill transfer from a virtual auscultation course to patients in a bedside scenario, possibly through the comparison of a virtual course with an auscultation simulator. The integration of bedside recorded heart sounds at multiple positions across the patient's thorax could improve the identification of typical patterns of sound radiation.

Once established, virtual auscultation courses are easily accessible and transferable to other faculties, upscaling the number of courses does not require additional equipment and they are not affected by contact restrictions. The presented course design employed peer teaching and may therefore be more resource-efficient than formats relying on post-graduate teaching staff. Moreover, they allow for training auscultation even in remote areas with limited access to alternative training possibilities.

#### 4.3. Limitations

Due to contact restrictions, the transfer of auscultation skills to patients in clinical practice has not been investigated in this study. However, good performance on an auscultation simulator correlated with good performance in real patients as shown by Fraser et al. [43] with regard to mitral regurgitation. Moreover, after training auscultation with virtual patients, auscultation performance on patients was equal to a control group participating in additional bedside teaching [17].

Long-time retention of auscultation skills was not assessed in this study. However, former studies have shown good long-term retention up to three years after training on an auscultation simulator [20], [21]. The sounds presented during and immediately after the VAC at the assessment T2 were the same. This might explain the superior performance of students participating in the VAC compared to SST simply by recognition of the previously presented heart sounds. In contrast, at T3 even though the same six pathologies were presented, the sound files were not identical with those at T2.

Moreover, even though also the VAC was supervised, the participant’s screens could not be monitored for data protection reasons. Thus, it cannot be ensured that participants worked with the literature provided or followed the virtual course the entire time.

The present study was sufficiently powered for valid conclusions, but more subtle effects of the VAC on auscultation performance with a moderate or small effect size may not have been detected.

## 5. Conclusion

This study demonstrates for the first time that training medical students with a virtual auscultation course via video conference may significantly improve their heart auscultation skills after one week on a simulator when compared to literature self-study. The digital teaching format was rated as good by the participants and lead to a self-assessed increase in competence and satisfaction. Thus, even after contact restrictions are lifted, an interactive online course may provide additional benefit and support acquiring fundamental skills in heart auscultation.

## Note

Bastian Malzkorn and Carsten Döing shared senior authorship.

## Competing interests

The authors declare that they have no competing interests. 

## Figures and Tables

**Table 1:**
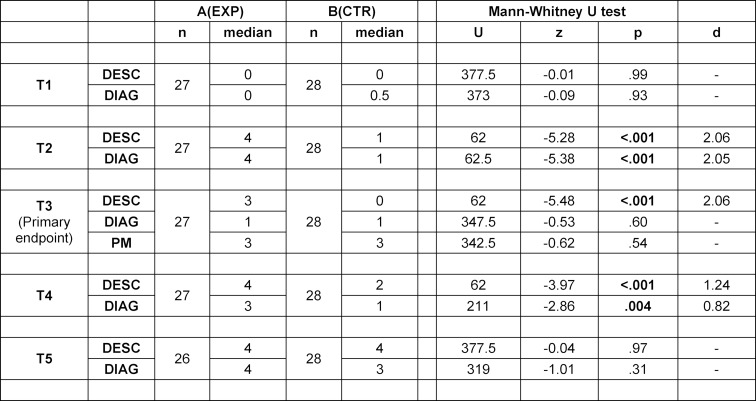
Cardiac auscultation performance (T1-T5): Sum scores of sound description (DESC) and diagnosis (DIAG) at T1-T5 as well as identification of the point of maximal intensity (PM) at T3 (maximum score=6). Performance of Mann-Whitney U tests comparing the experimental (A) and control group (B). Power (d) only sufficient for detecting a large effect size (power (1-β): d≥0.8=0.81).

**Table 2:**
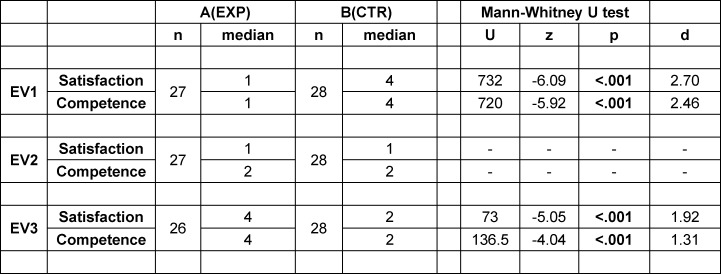
Course satisfaction and self-assessed competence: Six-point Likert scale-based evaluation (best score=1) of participant’s satisfaction and self-assessed increase in competence after course participation. Performance of Mann-Whitney U tests comparing the experimental (A) and control group (B) at EV1 and EV3. Power (d) only sufficient for detecting a large effect size (power (1-β): d≥0.8=0.81). No testing at EV2.
